# Non-Pharmacological Interventions for Reducing Fear and Anxiety in Patients Undergoing Third Molar Extraction under Local Anesthesia: Systematic Review and Meta-Analysis

**DOI:** 10.3390/ijerph191811162

**Published:** 2022-09-06

**Authors:** Natalie Sui Miu Wong, Andy Wai Kan Yeung, Kar Yan Li, Colman Patrick McGrath, Yiu Yan Leung

**Affiliations:** 1 Oral and Maxillofacial Surgery, Faculty of Dentistry, The University of Hong Kong, Hong Kong; 2 Applied Oral Sciences & Community Dental Care, Faculty of Dentistry, The University of Hong Kong, Hong Kong; 3 Clinical Research Centre, Faculty of Dentistry, The University of Hong Kong, Hong Kong

**Keywords:** non-pharmacological intervention, dental fear, dental anxiety, third molar, extraction

## Abstract

This report investigated the effectiveness of non-pharmacological interventions for reducing dental fear and anxiety in patients undergoing third molar extraction under local anesthesia. In November 2020, multiple electronic databases (Cochrane, EMBASE, MEDLINE, PsycInfo, PsycArticles, PubMed, and Web of Science) were searched for articles published in English. Inclusion criteria were randomized-controlled trials reporting the effectiveness of any non-pharmacological interventions in reducing fear or anxiety levels in patients with third molar extraction. A total of 3015 studies by electronic search and 2 studies by hand search were identified. After screening, 21 studies were eligible for systematic review. Seven studies were included in the meta-analysis. Study selection, data extraction, and quality assessment of the included studies were performed by two independent investigators. The anxiety levels after intervention in each study were pooled and meta-analyzed by the random-effect model. A significant reduction in anxiety level was observed in non-pharmacological intervention groups (SMD = −0.32; 95% CI −0.57 to −0.07; *p* = 0.01). Subgroup analyses showed that a significant anxiety reduction by non-pharmacological interventions could be demonstrated by pooled data from studies using psychometric assessments, but not from studies using physiological assessments. Non-pharmacological interventions appear to reduce fear and anxiety levels in patients undergoing third molar extraction under local anesthesia.

## 1. Introduction

Dental fear and anxiety are omnipresent, and there has been considerable interest in how to reduce dental fear and anxiety both by pharmacological and non-pharmacological interventions [1]. The terms dental fear, dental anxiety, and dental phobia are used interchangeably in the literature. They are known as psychological responses to un-comfortable or un-pleasant stimuli, but vary in the level of their severity. Fear is described as the immediate emotional response to an object, situation, or threat, whereas anxiety is defined as the anticipation of a future threat [2]. According to the *Diagnostic and Statistical Manual of Mental Disorders, Fifth Edition* (DSM-5) [3], phobia is defined as a type of anxiety disorder when an individual has an excessive level of fear towards a specific situation or object, which affects their daily functioning and quality of life. 

### 1.1. Dental Anxiety and Its Related Influences

Dental fear and anxiety are common phenomena in society and they have notable impacts on both patients and dental practitioners [4]. Prior studies suggested that people with dental anxiety tended to have a higher risk of experiencing oral disease, poor dental health, more missing teeth, and a higher prevalence of toothaches [5]. In addition, fear and anxiety can act as invisible barriers for patients seeking oral health services. Some obvious behavioral patterns include a higher tendency to delay their dental treatment, and a failure to show up for their dental appointments [6,7]. As a result, psychological well-being and social functioning among individuals with a high level of dental anxiety are commonly affected [8], such as sleep disturbance, embarrassment of poor oral health status, feelings of low self-esteem, and a decrease in both social interaction and participation in social activities [9]. As these psychosocial-related mental processes, behaviors, and the progressive worsening of un-treated oral health problems serve to reinforce and further intensify the level of fear, thus, a vicious cycle is established. Therefore, effective management of dental anxiety is desired for both patients and dental practitioners. 

### 1.2. Pharmacological and Non-Pharmacological Intervention in Reducing Fear in Dental Procedures 

A pharmacological approach is a traditional way to manage pain and dental anxiety; they are considered conventional treatments. Pharmacological interventions include pre-extraction medication, local anesthesia, and sedation. However, the use of drug-related methods may bring adverse effects in at-risk patients and patients with a medical history, such as nausea, dizziness, tachycardia, and other complications [10]. In regards to reducing undesirable emotional, physical, and behavioral responses of patients evoked by fear and anxiety, non-pharmacological interventions were applied as an adjunct in standard dental procedures. Existing approaches can be broadly categorized into psychological interventions (such as cognitive, behavioral, cognitive-behavior interventions, hypnosis, distraction and relaxation) [11,12,13,14], and other non-pharmacological interventions (such as enhanced pre-operative information, music listening, and virtual reality) [15,16,17,18,19].

### 1.3. Rationale and Objectives

With the high prevalence of dental fear worldwide [20], reducing levels of fear and anxiety in dental care procedures is desirable for both patients and dental practitioners. The use of non-pharmacological interventions as an adjunct to pharmacological treatments has been increasingly supported by recent systematic reviews [21,22,23]. However, most of the published reviews either address reducing anxiety in a general dental care procedure or focus exclusively on psychological interventions. There should be an analysis for third molar extraction, and it should cover non-pharmacological interventions as a whole. This is because third molar extraction is one of the most common clinical procedures in oral surgery, and it triggers fear the most when compared to other dental procedures or minor oral surgery procedures [24]. It was estimated that approximately every one in four dental patients had an impacted lower third molar [25]. Nearly one-quarter of them required general anesthesia or conscious sedation during the surgical removal of the third molar [26]. In general, the use of general anesthesia would increase the cost of medical treatments by at least 14% [27]. The economic burden on society could be effectively reduced if non-pharmacological interventions could reduce patients’ anxiety levels and, hence, switch them from undergoing general anesthesia to local anesthesia. Therefore, a comprehensive review focused particularly on the process of third molar extraction is essential. The present systematic review of randomized-controlled trials aimed to investigate the effectiveness of non-pharmacological interventions in reducing fear and anxiety in patients that are candidates for third molar extraction, and to compare the efficacy with or without non-pharmacological treatment as usual, or with local anesthesia alone in third molar extraction.

## 2. Materials and Methods

The study was reported according to the Preferred Reporting Items for Systematic Reviews and Meta-Analysis (PRISMA) guidelines [28]. It was not registered in PROSPERO.

### 2.1. Identification of Studies (PICOS) and Eligibility Criteria

The inclusion criteria were defined according to the PICOS framework. Patients (P)—patients undergoing third molar extraction aged 18 years or older were considered. Patients aged under 18 years were excluded, as the assessment measures were different for adults. Interventions (I)—any non-pharmacological intervention which aims to reduce stress, fear, or anxiety, implemented either before or during extraction procedures. Any non-pharmacological interventions that act as an adjunct to local anesthesia, with or without sedation, were included. Comparators (C)—all relevant control interventions were included, such as standard treatment care, treatment as usual (no intervention implemented), and placebo. Outcomes (O)—any physical or psychological outcomes explored were considered. Trials measuring mental state, stress, fear, or anxiety through validated assessment tools, including self-reported or observer-reported, were included. Trials that measured phobia were excluded, as phobia is a clinical diagnosis that affects and limits individuals’ daily functioning. Study design (S)—randomized-clinical trials were included in the systematic review, but only randomized-controlled trials were included in the meta-analysis.

### 2.2. Information Sources, Search Strategy and Study Selection

An electronic literature search was carried out in the databases Cochrane, EMBASE, MEDLINE, PsycInfo, PsycArticles, PubMed, and Web of Science. The search strategy is shown in Supplementary Materials File S1. The last search was performed in November 2020. Reference lists of relevant publications were screened. Titles and abstracts of eligible studies were reviewed by two investigators (NW and AY), independently. A hand search of the reference lists in included articles was performed in the second-round search. Disagreements were noted and resolved by discussion with a third investigator. 

### 2.3. Data Extraction

A pre-defined data extraction sheet was developed. Two investigators (NW and AY) independently reviewed the full text of the articles. One author performed data extraction and the second author checked the extracted data. Disagreements were resolved by discussion and reaching a consensus. Data from included studies were collected, which included: authors, publication year, journal, country, characteristics of the participants (including age and experiences of oral surgery), and characteristics of the trials (including sample size, number of surgeons involved in the study, type of intervention, group classification, time of intervention, the use of anesthesia, duration of surgery, measurement for variables, time for measurement, and major findings). 

### 2.4. Study Quality Assessment of Individual Studies

Quality assessment of the included studies was assessed by using the revised Cochrane risk-of-bias tool for randomized trials (RoB 2) [29] with five domains: (1) risk arising from the randomization process, (2) bias due to deviations from intended interventions, (3) bias due to missing outcome data, (4) bias in measurement of the outcome, and (5) bias in the selection of the reported result. The risk of bias assessment was conducted by two independent reviewers (NW and AY). Consensus was reached with discussion.

### 2.5. Summary Measures and Data Synthesis

The primary measure of the treatment effect was the reduction in fear and anxiety levels. Meta-analyses were performed using Review Manager (Version 5.4, The Cochrane Collaboration, 2020). Standardized mean difference (SMD) was used as the effect measure in the meta-analysis because different studies measured the outcome by the use of different psychometric or physiological assessment scales. Outcome data were analyzed using a random effect model with a 95% confidence interval (CI). Substantial heterogeneity between trials was assessed by the χ^2^ test with *p* < 0.10 and the I^2^ statistic of >50% [30]. If the above two criteria were not fulfilled, but the I^2^ statistic still ranged from 30% to 50%, then heterogeneity was still defined as moderate heterogeneity [30]. When the I^2^ statistic is <30%, heterogeneity might not be important.

### 2.6. Risk of Bias across Studies

Examination of publication bias was not performed, as only seven studies were eligible for meta-analysis [30].

## 3. Results

**Figure 1:**
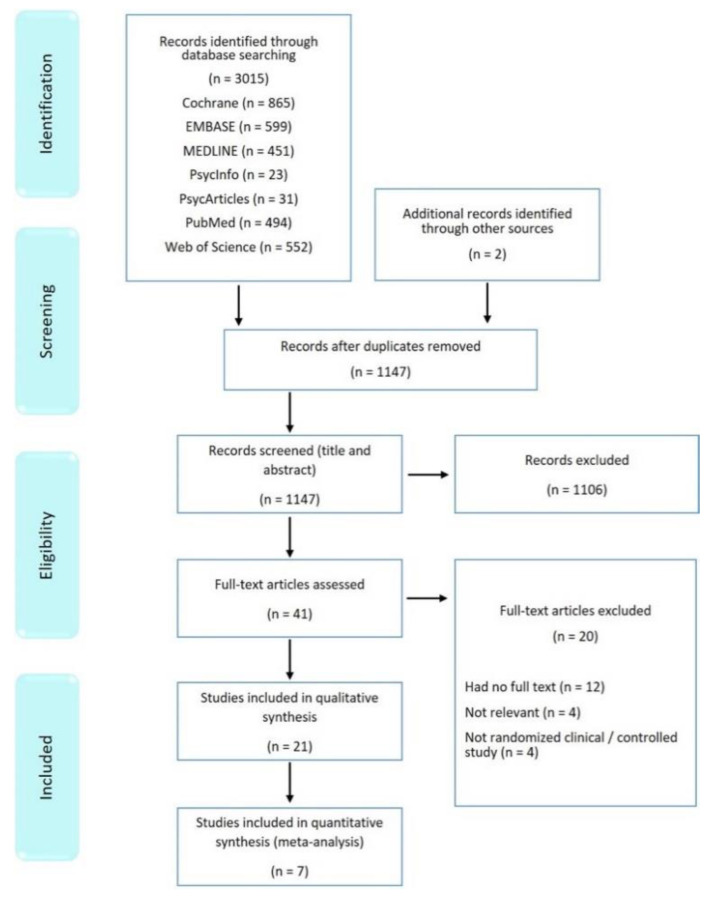
Flowchart of identification, screen, and assessing studies for inclusion eligibility.

Overall, 3015 potentially relevant articles were identified through database searches, and two more publications were identified through other sources. A total of 2976 articles were excluded after the removal of duplicates and abstract screening. A total of 41 publications were retrieved for full-text review. A total of 21 trials met the inclusion criteria and were included in the systematic review [13,15,19,31,32,33,34,35,36,37,38,39,40,41,42,43,44,45,46,47,48]. Figure 1 shows the flowchart of the study selection process recommended by the PRISMA Statement. The characteristics of the included studies are presented in Supplementary Table S1. Agreement between the two reviewers was reached following two phases: (1) title and abstract screening, and (2) full-text screening, with Kappa coefficient levels of agreement of 84.2% and 82.8%, respectively. 

### 3.1. Risk of Bias within Studies

**Figure 2:**
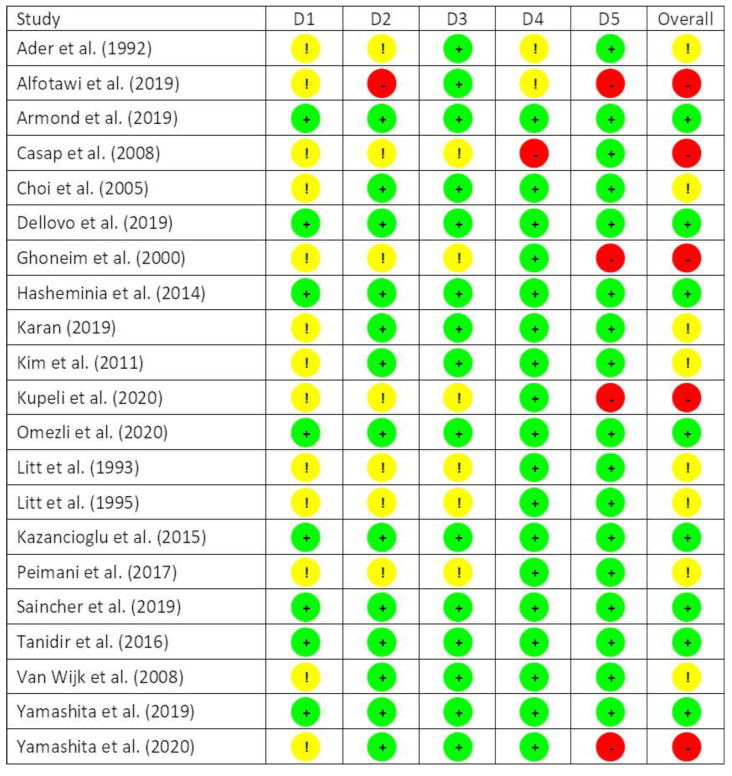
Quality assessment of included studies in a systematic review. Risk-of-bias judgments fall into three categories across five domains, with (+) being of low risk, (!) being of some concern, and (-) being of high risk. The five domains are as follows: (D1) bias arising from the randomization process; (D2) bias due to deviations from intended interventions; (D3) bias due to missing outcome data; (D4) bias in measurement of the outcome; and (D5) bias in the selection of the reported results [13,15,19,31,32,33,34,35,36,37,38,39,40,41,42,43,44,45,46,47,48].

The risk of bias varied across the 21 included publications. Eight studies were judged to be at low risk of bias; eight studies had some concerns, and the remaining five studies were at high risk of bias. The completed risk-of-bias assessments for each study are summarized in Figure 2.

### 3.2. Characteristics of Included Studies in Systematic Review

*General characteristics.* All studies included were published in English between 1992 and 2020 (Supplementary Table S1). The 21 trials were conducted in Turkey (*n* = 5), the US (*n* = 4), Brazil, Iran, Japan, and Korea (each *n* = 2), and India, Israel, the Netherlands, and Saudi Arabia (each *n* = 1). They were all randomized clinical trials published in scientific journals. A total of 15 of them were identified as randomized-controlled studies, and 6 of them were randomized clinical studies. Among those three randomized clinical studies, one was a split-mouth study, two were crossover studies, and three were clinical studies without a control group.

*Study participants.* The number of participants per study ranged from 16 to 300, with a total of 1849 study participants in the review. All studies recruited both males and females, except two studies were an all-female study and one study did not provide related information. The mean age of participants ranged from 22 to 35 years across all studies. Only seven studies reported previous oral surgery experiences of patients.

*Study design.* The most frequently investigated interventions were enhanced pre-operative information (*n* = 8) and music listening (*n* = 3) (Table 1). Meanwhile, two studies carried out multiple comparisons with different approaches, namely oral pre-medication, relaxation, self-efficacy enhancement, and intravenous needle desensitization. Interventions in 15 studies were applied before surgical procedures, while five interventions were applied during surgical procedures, and one study did not provide information. Among the 21 studies, the use of local anesthesia in surgical procedures was reported in 11 studies, the use of sedation was reported in 2 studies, and the use of anesthesia and sedation together was reported in 1 study. Only seven studies recorded the duration of the surgical procedure. The mean surgery time ranged from 16.32 to 41.4 min (Supplementary Table S1).

*Measurements.* Mental distress and anxiety of patients were primarily measured with patient-reported psychometric assessments and physiological assessments. Ten studies investigated anxiety with psychometric assessments only, one study investigated with physiological assessments only, and ten studies investigated with both assessments. The psychometric and physiological assessments employed among the 21 studies are listed in Table 2. The most frequently used psychometric and physiological measurements were state-trait anxiety inventory (STAI) and blood pressure (BP), respectively.

*Outcomes.* The primary outcome of all the studies was ‘changes of anxiety’ from baseline to after the implementation of non-pharmacological interventions (which could be applied before or during the third molar removal). Secondary outcomes were included in some studies, including pain level, analgesics used, satisfaction, episodes of vomiting, nausea, trismus, oedema, level of coping, self-efficacy, and other complications.

### 3.3. Results of Individual Studies and Syntheses of Results

**Figure 3:**
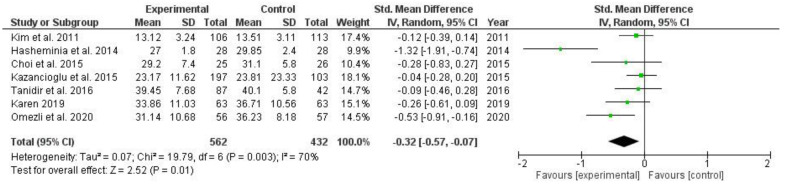
Forest plot of comparison: changes of anxiety levels in non-pharmacological interventions compared with control groups after third molar extraction [15,35,37,38,40,43,46].

The meta-analysis results were shown as forest plots of SMD and with a 95% CI. A total of fourteen studies were excluded. Thirteen studies were excluded because changes in the mean or SD were not available, and one of the studies was excluded because it did not have a control group. Studies presented with a high risk of bias were initially included, but no numerical data on these studies were available in the meta-analysis. Complete data were available for seven studies (Figure 3). Compared with all control groups, the anxiety level of the non-pharmacological intervention groups was reduced by 0.32 units. A significant reduction in anxiety level was found (SMD = −0.32; 95% CI −0.57 to −0.07; *p* = 0.01). The random effect model was applied, as substantial heterogeneity (*p* = 0.003; I^2^ = 70%) of the included studies was observed. One study was identified by retrospective exploration of heterogeneity and was excluded in second-round analyses. Low heterogeneity was detected (*p* = 0.36; I^2^ = 9%) after removing the study, and the finding was not affected (SMD = −0.18; 95% CI −0.32 to −0.04; *p* = 0.01). Anxiety levels were significantly decreased in non-pharmacological intervention groups compared with control groups.

### 3.4. Additional Subgroup Analyses

Multiple sub-group analyses were carried out, as timing of delivering non-pharmacological interventions varied among included studies. Some interventions were delivered before third molar extraction, while some were delivered during third molar extraction. In addition to the timing of intervention delivery, form of anxiety assessments was also considered as a factor that influences the result. Assessments of anxiety were categorized into psychometric assessments (i.e., self-administered questionnaires), such as Corah’s dental anxiety scale (DAS) and Kleinknecht’s dental fear survey (DFS), and physiological assessments (i.e., vital signs), such as heat rate (HR), blood pressure (BP), and respiratory rate (RR). Therefore, sub-group analyses were performed: (1) effectiveness of non-pharmacological interventions between groups with interventions applied before and during third molar extraction, and (2) effectiveness of non-pharmacological interventions between groups with psychometric and physiological assessment.

**Figure 4:**
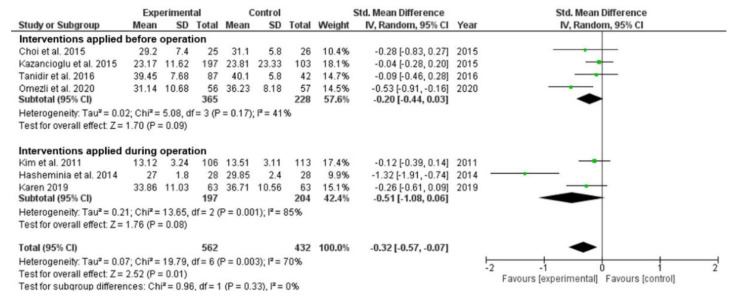
Forest plot of comparison: sub-group analyses—changes in anxiety level between groups with non-pharmacological interventions applied before and during third molar extraction [15,35,37,38,40,43,46].

*Interventions applied before* vs. *during third molar extraction.* Sub-group analyses were carried out. Among the seven included studies, non-pharmacological interventions were applied before third molar extraction in four studies, and during third molar extraction in three studies (Figure 4). The effect of applying non-pharmacological interventions before third molar extraction was not statistically significant (SMD = −0.20; 95% CI −0.44 to −0.03; *p* = 0.09). The heterogeneity of effect was moderate (*p* = 0.17; I^2^ = 41%). The effect of applying non-pharmacological interventions during third molar extraction was also not statistically significant (SMD = −0.51; 95% CI −1.08 to 0.06; *p* = 0.08). Heterogeneity was substantial (*p* = 0.001; I^2^ = 85%). After removing one identified distinctive study to reduce the heterogeneity, the finding was still not affected (SMD = −0.17; 95% CI −0.38 to 0.04; *p* = 0.11); no heterogeneity was detected (*p* = 0.53; I^2^ = 0%). The effect of applying non-pharmacological interventions during third molar extraction did not produce a statistically significant effect.

**Figure 5:**
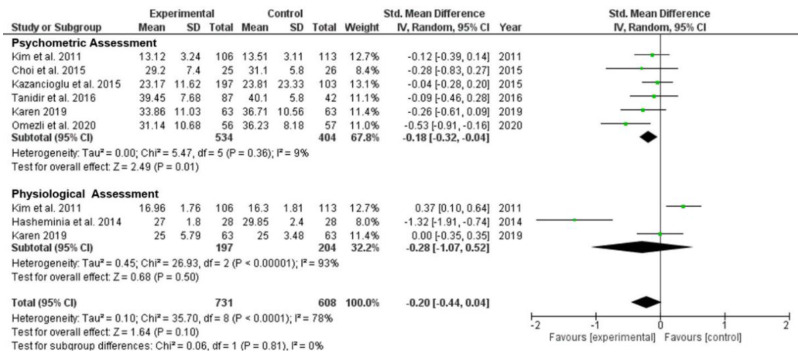
Forest plot of comparison: sub-group analyses—change in anxiety level between groups with psychometric assessments and physiological assessments after third molar extraction [15,35,37,38,40,43,46].

*Psychometric assessments* vs. *physiological assessments.* Comparison between psychometric and physiological assessments were carried out (Figure 5). Compared with all control groups, the data in six studies using psychometric assessments presented a significant reduction in anxiety levels (SMD = −0.18; 95% CI −0.32 to −0.04; *p* = 0.01). Low and possibly not important heterogeneity was detected (*p* = 0.36; I^2^ = 9%). However, data in three studies using physiological assessments did not produce a significant reduction in anxiety levels (SMD = −0.28; 95% CI −1.07 to 0.52; *p* = 0.50). Heterogeneity was substantial (*p* < 0.001; I^2^ = 93%) (Figure 5). The result was not affected after removing the identified distinctive study to reduce the heterogeneity (SMD = 0.20; 95% CI −0.16 to 0.56; *p* = 0.27). Heterogeneity was moderate (*p* = 0.10; I^2^ = 62%). Studies using physiological assessments did not identify significant reductions in dental anxiety.

## 4. Discussion

This meta-analysis identified that non-pharmacological interventions can generally reduce dental anxiety for patients undergoing third molar removal. This effect could not be shown when the studies were divided into interventions before and during third molar surgery. Sub-group analyses also demonstrated that the reduction in dental anxiety could be effectively reflected by psychometric but not physiological assessments. This finding added further insight to the theme of dental anxiety, as prior meta-analysis on non-pharmacological interventions for general dental treatment only reported an overall effect without distinguishing psychometric and physiological properties [21].

### 4.1. Inventory Used to Measure Dental Anxiety

It was found that the most frequently used inventory to measure dental anxiety was the state-trait anxiety inventory (STAI) [49], which is a scale for measuring the general current state of anxiety toward a situation or an event (i.e., state), and the tendency of an individual to experience anxiety symptoms (i.e., trait), but not specifically for measuring dental anxiety. Other inventories commonly recruited by the reviewed studies were dental anxiety specific, such as (Modified)/dental anxiety scale (MDAS/DAS) [50,51] and dental fear survey (DFS) [52]. However, these inventories were constructed without a theoretical basis. Most of these scales focus on specific dental stimuli only, while components, such as cognitive, behavioral, and physiological aspects were overlooked [2]. Additionally, these scales were criticized for poor construct validity [53], un-like the index of dental anxiety and fear (IDAF-4C^+^), a theoretically based psychological scale for measuring dental fear and anxiety [54].

Moreover, only two studies recruited patients with high dental anxiety according to the scores of the dental anxiety scale (DAS) or dental anxiety questionnaire (DAQ) (Supplementary Table S1), whereas other studies did not impose such an inclusion criterion. Lastly, no study involved a clinical psychologist to diagnose whether the recruited patients had anxiety or not.

### 4.2. Quality of Evidence

The risk of bias assessment showed that nearly a quarter (5/21 or 23.8%) of the reviewed studies were of high risk. This situation was more severe compared to a prior systematic review on non-pharmacological interventions for anxiety reduction in general dental procedures (10.3% high-risk studies) [21]. The high-risk studies mainly failed in domain D5 (bias in selection of the reported results). All five studies mentioned that they had recorded the anxiety levels by some rating scales and/or physiological data, such as heart rate and blood pressure, but did not provide a complete report in their results section (Figure 2). One study failed in domain D2 (bias due to deviations from intended interventions)—researchers switched two patients who refused to be in one intervention group to another intervention group without re-balancing the group compositions. This action caused deviations from the intended interventions without appropriate compensation. One study failed in domain D4 (bias due to measurement of the outcome), as it did not compare all outcomes with the control group.

### 4.3. Limitations

The number of eligible studies to be meta-analyzed was limited. It was because some studies reported whether the results were significant or not (*p*-values) only, whereas the actual data of mean and SD were not available. For instance, the sub-group meta-analysis on physiological assessments is composed of three studies only (Figure 5), rendering it difficult to ascertain the effectiveness of non-pharmacological interventions on them. Additionally, some studies presented the results in graphs or charts only, for which numerical data could not be extracted accurately and hence excluded from the meta-analysis. All of the above made it impossible to quantify the efficacy of each type of non-pharmacological intervention independently. Apart from these, readers should also be aware of the fact that anxiety is one of many mental disorders, such that it requires a medical diagnosis. It is inadequate to simply evaluate the (dental) anxiety level of a patient with a psychometric tool to be tallied by a researcher. Suspected cases need to be referred to a qualified psychiatrist or clinical psychologist who has the competency to correctly interpret the results and make a proper diagnosis. For instance, situation anxiety can be a manifestation of an underlying pathology that may be pharmaco-dependent. Meanwhile, some of the interventions reviewed can only be used in planned surgery but not in situations that involve emergency or urgent treatment, such as hypnosis or acupuncture.

### 4.4. Future Directions and Implications

Having had acute pain or an unpleasant dental experience in the past might condition a patient and make patients more anxious during dental treatments [4]. On the contrary, patients with prior tooth removal experience might experience less anxiety due to less un-certainty. Meanwhile, the working experience of the operating surgeons might affect the outcome, as inexperienced dentists might spend more time to complete the third molar surgery, potentially heightening the dental anxiety level of the patients [54]. The general state of health of patients, and the economic status of countries (hence, the resources allocated to the healthcare sector in general) may also affect the perception and anxiety level of patients. All these potentially influential factors were rarely reported in the studies reviewed in this report. In addition, some of the non-pharmacological interventions were applied several weeks before the day of operation. The duration between interventions applied and the day of surgery may become a factor influencing the efficacy of non-pharmacological interventions in reducing anxiety. Finally, the non-pharmacological interventions adopted in the reviewed studies were mainly operator-driven, with patients receiving them passively. With more attention paid to making dental patients more comfortable, more and more future studies should be expected to investigate various themes, such as comparing third molar surgery with conventional dental extraction [55], and evaluating upcoming modalities that are potentially anxiolytic, such as heartfulness meditation and virtual reality [56,57]. Future studies should consider the following two aspects: (1) applying more patient-driven interventions, such as biofeedback, for which patients would try to maintain their physiological measures at a baseline level (e.g., breathing rate, heart rate) by receiving visual or audio feedback; (2) an inventory with a more solid theoretical basis in psychology could be deployed to assess dental anxiety.

## 5. Conclusions

Within the limitations of this meta-analysis, non-pharmacological interventions appear to offer promising ways to alleviate dental anxiety for patients undergoing third molar removal. Their effects could be demonstrated by psychometric but not physiological assessments. Future studies should report more numerical data apart from statistical significance, as well as background information on both patients and surgeons in terms of prior dental experience, particularly with third molar surgery. These measures should improve the current issue that only a few eligible studies have been meta-analyzed.

## Figures and Tables

**Table 1:** Category of non-pharmacological interventions of included studies in a systematic review.

Interventions	*n*
Enhanced pre-operative information	8
Form of delivery (*n* = 7) Content of information (*n* = 1)	
Music listening	3
Smell/Odor	2
Hypnosis	2
Auriculotherapy	1
Acupuncture	1
Virtual reality	1
Separate day of consultation	1
Comparisons between oral pre-medication, relaxation, self-efficacy enhancement, and intravenous needle desensitization	2

**Table 2:** The use of measurement tools of included studies in systematic review.

Measurements	*n*
Psychometric Assessments	
State-Trait Anxiety Inventory (STAI)	10
Modified Dental Anxiety Scale (MDAS)	6
Dental Anxiety Scale (DAS)	5
Visual Analogue Scale (VAS)	5
Dental Fear Survey (DFS)	2
Numerical Rating Scale (NRS)	2
Patient Stress Response Index (PSRI)	2
Amsterdam Pre-operative Anxiety and Information Scale (APAIS)	1
Dental Anxiety Questionnaire (DAQ)	1
Short-Form Dental Anxiety Inventory (S-DAI)	1
Short Version of Fear of Dental Pain Questionnaire (S-FDPQ)	1
Physiological Assessments	
Blood Pressure (BP)	9
Heart Rate (HR)	7
Blood Oxygen Saturation (SpO_2_)	4
Respiratory Rate (RR)	3
Pulse Rate (PR)	2
Heart Rate Variability (HRV)	3
Electrodermal Activity (EDA)	1

## Data Availability

Data is available in the manuscript.

## Supplementary Materials

The following supporting information can be downloaded at: https://www.mdpi.com/article/10.3390/ijerph191811162/s1, File S1: Search strategy. Table S1: Characteristics of included studies in the systematic review. References [13,15,19,31,32,33,34,35,36,37,38,39,40,41,42,43,44,45,46,47,48] are cited in the Supplementary Materials.
