# Supplementary material for: Non-Pharmacological Interventions for Reducing Fear and Anxiety in Patients Undergoing Third Molar Extraction under Local Anesthesia: Systematic Review and Meta-Analysis
Source: Int J Environ Res Public Health. 2022 Sep 6;19(18):11162. doi: 10.3390/ijerph191811162 (PMC9517611; doi:10.3390/ijerph191811162)
Supplement: Supplementary file 1 [file ijerph-19-11162-s001.zip › Supplementary File S1.pdf]

File S1. Search strategy

PubMed

((Third Molar* OR Wisdom tooth OR wisdom teeth) AND (Extract* OR remov* OR exodontia)) AND (Behavior* OR conditioning OR cognitive OR cope OR coping OR Desensiti* OR Exposure OR flooding OR hypno* OR distract* OR reapprais* OR Mindfulness OR Meditation OR nonpharmacologic* OR non-pharmacologic* OR relax* OR breath* OR music* OR audi* OR visual* OR odor* OR smell* OR reinforce* OR tell show do OR tell-show-do OR model* OR muscle* OR acupuncture* OR biofeedback) AND (Clinical trial* OR controlled clinical trial* OR controlled trial* OR clinical controlled trial* OR Random*) AND (stress* OR anxi* OR fear* OR phobi* OR pain* OR emotion*)

Cochrane

((Third Molar* OR Wisdom tooth OR wisdom teeth) AND (Extract* OR remov* OR exodontia)) AND (Behavior* OR conditioning OR cognitive OR cope OR coping OR Desensiti* OR Exposure OR flooding OR hypno* OR distract* OR reapprais* OR Mindfulness OR Meditation OR nonpharmacologic* OR non-pharmacologic* OR relax* OR breath* OR music* OR audi* OR visual* OR odor* OR smell* OR reinforce* OR tell show do OR tell-show-do OR model* OR muscle* OR acupuncture* OR biofeedback) AND (Clinical trial* OR controlled clinical trial* OR controlled trial* OR clinical controlled trial* OR Random*) AND (stress* OR anxi* OR fear* OR phobi* OR pain* OR emotion*)

EMBASE

((Third Molar* or Wisdom tooth or wisdom teeth) and (Extract* or remov* or exodontia) and (Behavior* or conditioning or cognitive or cope or coping or Desensiti* or Exposure or flooding or hypno* or distract* or reapprais* or Mindfulness or Meditation or nonpharmacologic* or non-pharmacologic* or relax* or breath* or music* or audi* or visual* or odor* or smell* or reinforce* or tell show do or tell-show-do or model* or muscle* or acupuncture* or biofeedback) and (Clinical trial* or controlled clinical trial* or controlled trial* or clinical controlled trial* or Random*) and (stress* or anxi* or fear* or phobi* or pain* or emotion*))

MEDLINE

Adv search_All fields: ((“Third Molar*” OR “Wisdom tooth” OR “wisdom teeth”) AND (Extract* OR remov* OR exodontia)) AND (Behavior* OR conditioning OR cognitive OR cope OR coping OR Desensiti* OR Exposure OR flooding OR hypno* OR distract* OR reapprais* OR Mindfulness OR Meditation OR nonpharmacologic* OR non-pharmacologic* OR relax* OR breath* OR music* OR audi* OR visual* OR odor* OR smell* OR reinforce* OR “tell show do” OR “tell-show-do” OR model* OR muscle* OR acupuncture* OR “biofeedback”) AND (“Clinical trial*” OR “controlled clinical trial*” OR “controlled trial*” OR “clinical controlled trial*” OR Random*) AND (stress* OR anxi* OR fear* OR phobi* OR pain* OR emotion*)

PsycArticles

Searching Anywhere ((“Third Molar*” OR “Wisdom tooth” OR “wisdom teeth”) AND (Extract* OR remov* OR exodontia)) AND (Behavior* OR conditioning OR cognitive OR cope OR coping OR Desensiti* OR Exposure OR flooding OR hypno* OR distract* OR reapprais* OR Mindfulness OR Meditation OR nonpharmacologic* OR non-pharmacologic* OR relax* OR breath* OR music* OR audi* OR visual* OR odor* OR smell* OR reinforce* OR “tell show do” OR “tell-show-do” OR model* OR muscle* OR acupuncture* OR “biofeedback”) AND (“Clinical trial*” OR “controlled clinical trial*” OR “controlled trial*” OR “clinical controlled trial*” OR Random*) AND (stress* OR anxi* OR fear* OR phobi* OR pain* OR emotion*)

PsycInfo

Search Anywhere ((“Third Molar*” OR “Wisdom tooth” OR “wisdom teeth”) AND (Extract* OR remov* OR exodontia)) AND (Behavior* OR conditioning OR cognitive OR cope OR coping OR Desensiti* OR Exposure OR flooding OR hypno* OR distract* OR reapprais* OR Mindfulness OR Meditation OR nonpharmacologic* OR non-pharmacologic* OR relax* OR breath* OR music* OR audi* OR visual* OR odor* OR smell* OR reinforce* OR “tell show do” OR “tell-show-do” OR model* OR muscle* OR acupuncture* OR “biofeedback”) AND (“Clinical trial*” OR “controlled clinical trial*” OR “controlled trial*” OR “clinical controlled trial*” OR Random*) AND (stress* OR anxi* OR fear* OR phobi* OR pain* OR emotion*)

Web of Science

(Basic search) All fields: ((("Third Molar*" OR "Wisdom tooth" OR "wisdom teeth") AND (Extract* OR remov* OR exodontia))) AND All fields: ((Behavior* OR conditioning OR cognitive OR cope OR coping OR Desensiti* OR Exposure OR flooding OR hypno* OR distract* OR reapprais* OR Mindfulness OR Meditation OR nonpharmacologic* OR non-pharmacologic* OR relax* OR breath* OR music* OR audi* OR visual* OR odor* OR smell* OR reinforce* OR "tell show do" OR "tell-show-do" OR model* OR muscle* OR acupuncture* OR "biofeedback")) AND All fields: (("Clinical trial*" OR "controlled clinical trial*" OR "controlled trial*" OR "clinical controlled trial*" OR Random*)) AND All fields: ((stress* OR anxi* OR fear* OR phobi* OR pain* OR emotion*))
