# Supplementary material for: Non-Pharmacological Interventions for Reducing Fear and Anxiety in Patients Undergoing Third Molar Extraction under Local Anesthesia: Systematic Review and Meta-Analysis
Source: Int J Environ Res Public Health. 2022 Sep 6;19(18):11162. doi: 10.3390/ijerph191811162 (PMC9517611; doi:10.3390/ijerph191811162)
Supplement: Supplementary file 1 [file ijerph-19-11162-s001.zip › Supplementary Table S1.pdf]

Supplementary Table S1. Characteristics of included studies in systematic review [13,15,19,32–49].

Authors (Years) / Country	Sample size	Mean age	Previous oral surgery exp.	Outcome	Category of Intervention	Groups		Time of Intervention	Anaesthesia	No. of surgeon	Duration of Surgery	Psychometric Assessment of Anxiety [a]	Physiological Assessment of Anxiety [b]	Time of Measurements
						Study group	Control group							
Ader et al. (1992) / US	35<br>M (16)<br>F (19)	24.4	--	(1) Anxiety<br>(2) Satisfaction<br>(3) Amount learned	Enhanced preoperative information received	Group 1: Interactive video (disc group)<br><br>Group 2: Noninteractive video (tape group)	N/A	Before surgery<br>At the time of consultation (few weeks before surgery)	--	--	--	(a) STAI	N/A	(a) Before and after intervention, before surgery (several weeks after intervention)
Alfotawi et al. (2019) / Saudi Arabia	40<br>F (40)	--	--	(1) Anxiety	Enhanced preoperative information received	Group 1: Verbal instruction<br><br>Group 2: Audio-visual instruction	N/A	--	LA	--	--	(a) MDAS<br>(b) 0-5 Scale	(c) HR	(a) Immediately before surgery<br>(b) and (c) 8 different time point: on dental chair, after instructions, after injection, before extraction, drilling, after extraction, suturing, before leaving
Armond et al. (2019) / Brazil	17<br>M (3)<br>F (14)	22.5	--	(1) Anxiety<br>(2) Pain<br>(3) Oedema<br>(4) Trismus	Acupuncture	Acupuncture	Placebo	Before surgery:<br>Four sessions for each side (min. interval of 45 days): 30 minutes before surgery, 24,48,72 hours after surgery	LA	Same surgeon for both sides	?	(a) STAI<br>(b) VAS	N/A	(a) and (b) First evaluation, before and after intervention, and immediately before surgery
*Casap et al. (2008) / Israel	60	--	--	(1) Anxiety	Enhanced preoperative information received	Group 1: Detailed informed consent<br><br>Group 2: Simplified informed consent	Standard preparation as usual	Before surgery	--	--	--	N/A	(a) PR<br>(b) BP<br>(c) EDA	(a) and (b) Before and after intervention<br>(c) Before, during, and after intervention
*Choi et al. (2015) / Korea	51<br>M (41)<br>F (10)	22.4	Yes (9)<br>No (42)	(1) Anxiety<br>(2) Amount learned / Understanding	Enhanced preoperative information received	Written consent + Audio-visual slideshow (PPT)	Standard preparation as usual (Written consent + verbal explanation)	Before surgery	--	2	--	(a) STAI<br>(b) DAS<br>(c) VAS	N/A	(a), (b) and (c) Immediately before surgery and one week after surgery
Dellovo et al. (2019) / Brazil	30<br>M (?)<br>F (?)	M (35)<br>F (28)	--	(1) Anxiety	Auriculotherapy *vs.* Midazolam	Group 1: medication + placebo auriculotherapy<br><br>Group 2: Placebo medication + auriculotherapy	N/A	Before surgery<br><br>Medication / Placebo: 30 mins before surgery;<br>Auriculotherapy / Placebo auriculotherapy: 5 days before surgery<br><br>(Two sessions of surgery, minimum interval of 15 days)	LA	--	--	(a) DAS	(b) BP<br>(c) HR<br>(d) SpO_2	(a) 1 week before surgery (baseline), immediately before surgery, and follow up visit<br>(b), (c) and (d): 1 week before surgery (baseline), 30 mins after drug administration, after local anaesthesia, incision, tooth removal, and suture

Table S1. Characteristics of included studies in systematic review. (CONT')

Authors (Years) / Country	Sample size	Mean age	Previous oral surgery exp.	Outcome	Category of Intervention	Groups		Time of Intervention	Anaesthesia	No. of surgeon	Duration of Surgery	Psychometric Assessment of Anxiety	Physiological Assessment of Anxiety	Time of Measurements
						**Study group**	**Control group**							
*Ghoneim et al. (2000) / US	60<br>M (25)<br>F (35)	C (24)<br>S (23)	--	(1) Anxiety<br>(2) Nausea<br>(3) Pain<br>(4) Analgesics used<br>(5) Episodes of vomiting<br>(6) Complication	Hypnosis	With hypnotic induction audio tape	No hypnotic induction audio tape	Before surgery (Every day for one week before surgery)	(a) Sedation<br>(b) LA	1	Control group: 19.7 mins<br><br>Study group 1: 20.3 mins	(a) STAI	(b) BP<br>(c) HR	(a) One week before treatment and surgery (baseline), immediate before and after surgery, first 3 days after surgery<br>(b) and (c) Not mentioned
*Hasheminia et al. (2014) / Iran	56<br>M (31)<br>F (25)	C (28)<br>S (26)	--	(1) Anxiety	Smell / Odor	With fragrance	No fragrance	During surgery	LA	1	20-25 mins for each surgery	(a) DAS (Used to determine the eligibility of patients)	(b) BP<br>(c) PR<br>(d) RR	(a) Before surgery<br>(b), (c), and (d) Twice before entered the waiting room, before anaesthesia induction, immediately before surgery, and the end of treatment session
*Karan (2019) / Turkey	126<br>M (30)<br>F (96)	--	Yes (48)<br>No (78)	(1) Anxiety<br>(2) Pain<br>(3) Satisfaction	Smell / Odor	With lavender oil	No lavender oil	Before surgery (In a separate room for 3 minutes prior to surgery)	LA	--	16.32 – 16.78 mins	(a) DAQ (Used to determine the eligibility of patients)<br>(b) MDAS<br>(c) STAI-S	(d) SBP<br>(e) DBP<br>(f) RR<br>(g) HR<br>(h) SpO_2	(a), (b) and (c) Before and after surgery<br><br>(d), (e), (f) and (g) Before surgery, during, and after surgery
*Kim et al. (2011) / Korea	219<br>M (122)<br>F (97)	--	Yes (106)<br>No (113)	(1) Anxiety<br>(2) Pain	Music	With music	No music	During surgery	LA	2	22.8 ± 5.71 mins	(a) DAS	(b) SBP<br>(c) DBP<br>(d) HR<br>(e) RR	(a) Before surgery, 20 mins after surgery<br>(b), (c), and (d) Arrival at operation room, beginning of surgery, middle of surgery, and end of surgery
*Kupeli et al. (2020) / Turkey	80<br>M (36)<br>F (44)	24.1	--	(1) Anxiety	Music	Group 1: Turkish music;<br>Group 2: Classical music of Western culture;<br>Group 3: Soft rock music	No music	During surgery	LA	1	--	(a) DAS	(b) BP<br>(c) HR<br>(d) SpO_2	(a), (b), (c) and (d) On dental chair before music treatment, 5 minutes of music listening, recorded at 5-minute intervals after anesthesia, and after surgery
*Omezli et al. (2020) / Turkey	113<br>M (31)<br>F (82)	23	No	(1) Anxiety<br>(2) Pain	Enhanced preoperative information received	Verbal information + written document + video	Standard preparation as usual (Verbal information about + written document	Before surgery	--	1	--	(a) MDAS<br>(b) APAIS<br>(c) STAI-S<br>(d) STAI-T	N/A	(a), (b), (c) 1 week before surgery, after the consent process, immediately after surgery and one week after surgery

Table S1. Characteristics of included studies in systematic review. (CONT').

Authors (Years) / Country	Sample size	Mean age	Previous oral surgery exp.	Outcome	Category of Intervention	Groups		Time of Intervention	Anaesthesia	No. of surgeon	Duration of Surgery	Psychometric Assessment of Anxiety	Physiological Assessment of Anxiety	Time of Measurements
						Study group	Control group							
*Litt et al. (1993) / US	70<br>M (27)<br>F (43)	26.2	No		Multiple comparisons: Oral premedication (OP), relaxation (RE), and self-efficacy enhancement (SE), and intravenous needle desensitization	Group 1: OP;<br>Group 2: RE;<br>Group 3 SE	Standard preparation + Attention	Before surgery	Intravenous sedation	--	--	(a) DFS<br>(b) VAS<br>(c) PSRI	N/A	(a) Before intervention<br>(b) After intervention and immediately after surgery<br>(c) Immediately after surgery
*Litt et al. (1995) / US	231<br>M (77)<br>F (154)	24.9	No		Multiple comparisons: Oral premedication (OP), relaxation (RE), self-efficacy enhancement (SE), and intravenous needle desensitization (IV)	Group 1: OP;<br>Group 2: RE;<br>Group 3: SE;<br>Group 4: IV	Standard preparation + Attention	Before surgery	Intravenous sedation	--	--	(a) DFS<br>(b) VAS<br>(c) PSRI	N/A	(a) Consultation stage (baseline)<br>(b) After intervention (i.e. before surgery and immediately after surgery<br>(c) Immediately after surgery
*Kazancioglu et al. (2015) / Turkey	300<br>M (140)<br>F (160)	22.6	--	(1) Anxiety<br>(2) Pain	Enhanced preoperative information received	Study group 1: Verbal group, Basic information (verbally) + information of operative procedures and recovery (verbally)<br><br>Study group 2: Video group, Basic information (verbally) + information of operative procedures and recovery (video)	Standard preparation	Before surgery	LA	1	Control group: 22±14 mins<br><br>Study group 1: 25±11 mins<br><br>Study group 2: 26±01 mins	(a) DAS<br>(b) STAI-S<br>(c) STAI-T	N/A	(a) and (b) Before surgery, immediately after surgery, one week after surgery<br><br>(c) Before surgery
Peimani et al. (2017) / Iran	16<br>M (6)<br>F (10)	22	--	(1) Anxiety<br>(2) Pain	Hypnosis and Local anesthesia	Group 1: Hypnotized in the 1st session and received anesthetic medication in the 2nd session<br><br>Group 2: Reverse order of group 1	N/A	Before surgery<br><br>(Two sessions of surgery, minimum interval of 1 week)	N/A	--	--	(a) STAI	N/A	(a) Before and after treatment
Saincher et al. (2019) / India	26<br>M (10)<br>F (16)	Group 1: 25.5<br><br>Group 2: 23.3	--	(1) Anxiety	Enhanced preoperative information received	Group 1: Verbal group<br><br>Group 2: Video group	--	Before surgery	LA	1	--	(a) MDAS	(b) HR<br>(c) SpO_2<br>(d) SBP<br>(e) DBP	(a) Before surgery<br>(b) and (c) In waiting area, on the dental chair, incision, bone drilling, tooth elevation, suturing, and in the postoperative period<br>(d) 15 min before and 15 min after surgery

Table S1. Characteristics of included studies in systematic review. (CONT')

Authors (Years) / Country	Sample size	Mean age	Previous oral surgery exp.	Outcome	Category of Intervention	Groups		Time of Intervention	Anaesthesia	No. of surgeon	Duration of Surgery	Psychometric Assessment of Anxiety	Physiological Assessment of Anxiety	Time of Measurements
						Study group	Control group							
*Tanidir et al. (2016) / Turkey	129<br>M (49)<br>F (80)	--	Yes (29)<br>No (100)	(1) Anxiety<br>(2) Pain	Enhanced preoperative information received	Group 1: Dubbed video<br><br>Group 2: Silent video	Without video	Before surgery	--	--	--	(a) DAQ<br>(b) STAI-T<br>(c) STAI-S<br>(d) MDAS	N/A	(a), (b), and (c) 3 days before surgery (1st visit), after intervention (2nd visit), before surgery (3rd visit) and 24 hours after surgery (4th visit)
*van Wijk et al. (2008) / Netherland	50<br>M (22)<br>F (28)	M (26)<br>F (25)	--	(1) Anxiety<br>(2) Pain	Effect of separate consultation before surgery	Separated of consultation and surgical day	Consultation and surgery on the same day	Before surgery (At the time of consultation)	--	1	--	(a) NRS<br>(b) S-DAI	N/A	(a) and (b) Before surgery and one week after surgery
*Yamashita et al. (2019) / Japan	40<br>M (0)<br>F (40)	27.7	--	(1) Anxiety	Music	With Music	Without music	During surgery	LA	1	Control group: 35.7±9.7 mins<br><br>Music group: 41.4±11.1 mins<br><br>Total: 38.6±10.7 mins	(a) MDAS<br>(b) STAI-S<br>(c) STAI-T	(d) HRV<br>(e) SBP	(a) and (b) Before and after surgery<br>(c) Every 2 seconds during surgery<br>(d) Every 2 minutes during surgery
*Yamashita et al. (2020) / Japan	100<br>M (40)<br>F (60)	Control (35)<br>Study (27.6)	--	(1) Anxiety<br>(2) Pain<br>(3) Satisfaction	Virtual Reality (VR)	With VR	Without VR	During surgery	LA	1	--	(a) VAS	(b) HRV	(a) Before and after intervention<br>(b) Before intervention and 10 minutes after the start of surgery

"--" = Not mentioned; "?" = Not clear; F = Female; M = Male; LA = Local anaesthesia; N/A = Not applicable

* Studies are randomized controlled trials. Studies without '*' are randomized clinical trials

[a] APAIS =Amsterdam Preoperative Anxiety and Information Scale; DAQ = Dental Anxiety Questionnaire; DAS = Dental Anxiety Scale; DFS = Dental Fear Survey; MDAS = Modified Dental Anxiety Scale; NRS = Numerical rating scale; PSRI = Patient Stress Response Index; S-DAI = Short form Dental Anxiety Inventory; STAI = State-Trait Anxiety Inventory; VAS = Visual Analogue Scale

[b] BP = Blood pressure; EDA = Electrodermal Activity; HR = Heart rate; HRV = Heart rate variability; PR = Pulse rate; RR = Respiratory rate; SpO_2 = Blood oxygen saturation
